# Usefulness of ultrasonography for dynamic evaluation of medial meniscus hoop function in early knee osteoarthritis

**DOI:** 10.1038/s41598-021-99576-3

**Published:** 2021-10-11

**Authors:** Kengo Shimozaki, Junsuke Nakase, Kazuki Asai, Rikuto Yoshimizu, Mitsuhiro Kimura, Tomoyuki Kanayama, Takashi Kitagawa, Hiroyuki Tsuchiya

**Affiliations:** 1 Department of Orthopedic Surgery, Graduate School of Medical Sciences, Kanazawa University, 13-1 Takara-machi, Kanazawa-shi, Ishikawa-ken 920-8641 Japan; 2 Department of Physical Therapy, School of Health Sciences, Shinshu University, Matsumoto, Japan

**Keywords:** Medical research, Musculoskeletal system

## Abstract

This study aimed to evaluate the dynamics of the medial meniscus during knee flexion–extension by ultrasonography and compare them with MRI findings to confirm the usefulness of ultrasonography for evaluating early knee osteoarthritis (KOA). In total, 100 patients were diagnosed with early KOA using clinical and radiographical findings. Dynamic ultrasonographic evaluation and MRI were performed in all patients. Medial meniscal extrusion (MME) and medial meniscal tears were evaluated via ultrasonography and MRI. Abnormal MME was defined as MME > 2 mm on ultrasonography during knee extension. Patients with abnormal MME were divided into two groups: a decrease group (group D) and a non-decrease group (group N). Age, sex, absence or type of meniscus tear, and MME were compared between the two groups. Of the 100 patients, 75 demonstrated MME > 2 mm at knee extension. MME at all assessment positions using ultrasonography and MRI were significantly greater in group N (n = 34) than that in group D (n = 41). Medial meniscus posterior root tears or radial tears were observed in most cases in group N. A lack of decrease in MME from 0° to 90° of flexion on ultrasonography was a characteristic finding in patients with a loss of meniscal hoop function.

## Introduction

Knee osteoarthritis (KOA) is a representative cause of disability in older people worldwide^1^. To date, there are 27 million symptomatic adults with KOA in the USA and over 8 million in Japan, and these numbers continue to increase^2,3^. Furthermore, KOA begins from the medial side in many Japanese patients^3^. Given that progression of KOA may necessitate surgical treatment, such as total knee arthroplasty or osteotomies around the knee^4^, early diagnosis and intervention have attracted scientific attention in recent years^5^. Although its etiology remains to be thoroughly investigated, previous studies have reported that the prevalence of early KOA is 9.5% in men and 15.0% in women, with the highest prevalence occurring among middle-aged women in Japan^6^.

**Figure 1:**
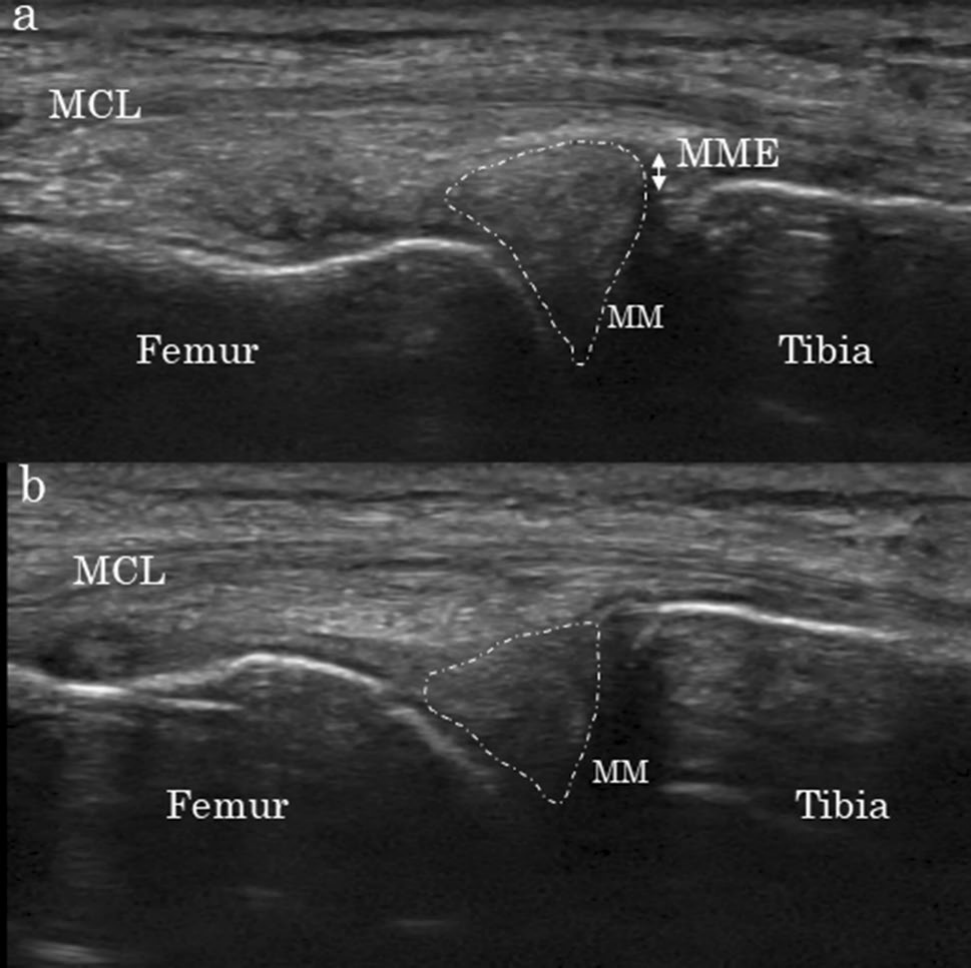
Change in MME from a knee flexion angle of 0° (**a**) to 90° (**b**) in a healthy adult. The medial meniscus that is slightly extruded at a knee flexion angle of 0° is retracted into the joint by knee flexion, at which point the extrusion disappears. MM is indicated by the dotted line. *MCL* medial collateral ligament, *MME* medial meniscal extrusion, *MM* medial meniscus.

Early KOA does not present with the characteristic clinical symptoms or radiographic signs of established KOA. Therefore, researchers have reported that magnetic resonance imaging (MRI) and arthroscopy are useful in detecting the full spectrum of pathological changes within the joint tissue^5^. Although our current understanding suggests that MRI can be used to detect early KOA^7,8^, this method has been excluded from the diagnostic criteria for early KOA, as it is costly and cannot be frequently used^9,10^. Therefore, in the present study, we focused on the efficiency of ultrasonography in identifying patients with early KOA, as the technique is simple and widely available in clinical settings. Because previous studies have already reported the usefulness of ultrasonography for meniscal evaluation, our novel dynamic ultrasonographic evaluation focused on the medial meniscus, where medial KOA findings are known to appear early^8,11,12^. Indeed, dynamic evaluation represents the strongest point of ultrasonography. For example, Fig. 1 shows dynamic ultrasonography findings in a healthy adult with a mildly extruded meniscus during knee extension and a corresponding decrease during knee flexion. The medial meniscus has been reported to undergo backward translation due to knee flexion^11^, which can be detected via ultrasonography as medial meniscal movement into the joint when the hoop function of the medial meniscus is normal. In contrast, longitudinal and degenerative tears retain some hoop function, while complete radial tear leads to complete loss of meniscal hoop function^13,14^. In the loss of hoop function cases with early medial KOA, this meniscal dynamic movement during knee flexion using ultrasonography may be absent.

Many ultrasonography studies have evaluated the state of the meniscus with or without load conditions in both healthy individuals^15^ and patients with moderate or severe KOA^16,17^. However, to the best of our knowledge, no study has evaluated patients exhibiting Kellgren–Lawrence (K–L) grades of 0 or 1. Moreover, the relationship between medial meniscal function and medial meniscal extrusion (MME) under loading conditions remains unknown.

Given the abovementioned setting, the present study aimed to evaluate the dynamics of the medial meniscus during knee flexion–extension via ultrasonography and compare them with MRI findings. Our goal was to confirm the usefulness of ultrasonography for evaluating early medial KOA. We hypothesized that dynamic ultrasonographic evaluation would be useful in assessing medial meniscal function in patients with early medial KOA, which may in turn enable appropriate additional examinations, early diagnosis, and intervention.

## Results

Of the 100 patients (average age: 63.3 ± 9.8 years, age range: 49–83 years), 61 were women and 39 were men. Moreover, ultrasonography revealed that 75 patients (75%) had MME > 2 mm at 0° of knee flexion. In other words, 25 patients (25%) had MME ≤ 2 mm at 0° of knee flexion, and the average MME at 0° of knee flexion was 1.2 ± 0.4 mm, which was almost normal^15^. The correlation coefficient between MRI and ultrasonography measurements of MME was r = 0.8, indicating a very strong positive correlation. The decrease group (Group D) included 41 patients (25 women and 16 men), while the non-decrease group (Group N) included 34 patients (26 women and 8 men). From 0° to 90° of knee flexion, MME values decreased by 1.8 ± 0.8 mm in group D and 0.4 ± 0.3 mm in group N. MME decreased significantly from 0° to 90° of knee flexion in both groups (*P* < 0.01), and the absolute decrease in MME from 0° to 90° of knee flexion differed significantly between the two groups (*P* < 0.01), according to the two-way analysis of variance. Ultrasonography revealed that MME at 0° and 90° of knee flexion was significantly greater in group N than in group D. Similarly, MRI also revealed significantly higher MME in group N than in group D (Table 1). For ultrasonographic assessment, the intra-class correlation coefficients under knee flexions at 0° and 90° were 0.908 and 0.898, respectively.

**Table 1:** MRI and ultrasonography measurements of MME in the two groups.

	Group D (n = 41)	Group N (n = 34)	*P* value
Age (years)	61.6 ± 9.5	64.6 ± 9.5	0.17
Sex (women:men)	25:16	26:8	0.21
MME by MRI (mm)	2.5 ± 1.4	3.6 ± 1.4	< 0.01
MME by ultrasonography at knee flexion of 0 degrees (mm)	3.0 ± 1.0	3.6 ± 1.0	0.014
MME by ultrasonography at knee flexion of 90 degrees (mm)	1.1 ± 1.0	3.2 ± 1.1	< 0.01
Change in MME from 0° to 90° of knee flexion (mm)	1.8 ± 0.8	0.4 ± 0.3	< 0.01

*MME* medial meniscal extrusion, *MRI* magnetic resonance imaging.

In group D, 25 horizontal or flap tears and four longitudinal tears were observed on MRI, and 11 cases exhibited only degenerative changes; however, there was only one case of posterior root tear. In contrast, in group N, MRI revealed 24 medial meniscus posterior root tears and three radial tears, which were directly linked to the deterioration of hoop function (Table 2). MRI revealed changes in the medial meniscus as well as osteoarthritis changes (e.g., osteophyte formation), although there were no findings suggestive of other inflammatory diseases. For MRI assessments, the intra-class correlation coefficient and inter-class reliability values were 0.911 and 0.889, respectively.

**Table 2:** Medial meniscus injury formation detected via magnetic resonance imaging.

	Group D (n = 41)	Group N (n = 34)
No tear or only degenerative changes	11 (26.8%)	2 (5.9%)
Horizontal or flap tear	25 (60.9%)	5 (14.7%)
Longitudinal tear	4 (9.7%)	0 (0%)
Radial tear without posterior root tear	0 (0%)	3 (8.8%)
Posterior root tear	1 (2.4%)	24 (70.6%)

## Discussion

In this study, we compared the dynamics of the medial meniscus during knee flexion–extension, as measured using ultrasonography. Most importantly, our analysis revealed that MME > 2 mm occurred in 75% of early medial KOA cases. Additionally, ultrasonography was able to identify a lack of decrease in MME from 0° to 90° of flexion, indicating a loss of medial meniscus hoop function.

Although KOA findings have been reported to originate from damage to the cartilage or meniscus^18^, osteophytes^19^, or bone marrow lesions^20^, these assertions remain controversial. In the present study, ultrasonography revealed that 75% of patients with early medial KOA had abnormal MME, which cannot be detected by radiography. These results suggest that many patients with early medial KOA exhibit abnormal meniscal findings at the first consultation. In contrast, some patients had no osteophytes on X-ray or meniscal injuries on MRI despite an abnormal MME, suggesting that dysfunction of the meniscus caused KOA in these cases.

Several studies have reported that medial meniscus posterior root or radial tears can lead to a loss of function in the meniscal hoop, which disperses axial loads into hoop stresses during loading^21–23^. Another biomechanical study demonstrated that medial meniscus posterior root tears can be functionally and biomechanically comparable with the outcome of total meniscectomy^21^. Furthermore, these meniscus tears increase articular cartilage contact pressure, accelerate degenerative changes^24^, and ultimately lead to spontaneous osteonecrosis of the knee, severe KOA, and hemi- or total knee arthroplasty^25,26^. Early diagnosis of these meniscus injuries, especially medial meniscus posterior root tears, is important because early surgical and/or physical interventions are necessary to avoid KOA progression^27,28^. However, as these meniscus tears could only be diagnosed by MRI until now, many cases may have been overlooked, as MRI has not been performed in all patients with early medial KOA. In our study, MME remained unchanged from extension to flexion of the knee in patients with medial meniscus posterior root tears, in accordance with the results of an MRI-based study by Masuda et al.^29^. Moreover, similar findings were successfully demonstrated by ultrasonography in the present study. The use of dynamic ultrasonography to evaluate meniscal hoop function during screening may allow patients to undergo MRI at an appropriate time, which would in turn aid in determining the most appropriate early interventions, including surgery. Furthermore, considering that approximately 70% cases of group N were of medial meniscus posterior root tears, this ultrasonographic evaluation method may lead to a specific detection tool for medial meniscus posterior tear, and we plan to conduct further research focusing on medial meniscus posterior root tear in the future.

In contrast, it has been reported that the medial meniscus has some hoop function even after longitudinal tear or partial meniscectomy, while the hoop function disappears with a complete radial tear, included in the medial meniscus posterior root tear^13,14^. Furthermore, cartilage degeneration progresses more slowly in degenerative tear cases than in radial tear cases^30^, suggesting that meniscal hoop function remains in degenerative tears contrary to radial tears. In this study, it is considered that the MME decreased because of knee flexion because the hoop function was maintained in degenerative tear cases, which were abundant in group D. However, even for some cases in which MME did not decrease from 0° to 90° of knee flexion (group N), meniscus tears could not be found. This finding may be related to meniscal degeneration. Strong degeneration of the meniscus is thought to cause not only meniscal tearing but also meniscal dysfunction leading to extrusion^31^. The extent of meniscal degeneration is thought to be affected by the duration of KOA, lower limb alignment, and tightness of the soft tissue around the knee. Thus, assessments of KOA duration and lower limb alignment are required in the future.

A distinct strength of the current study was the use of dynamic ultrasonography to evaluate the medial meniscus. Our results provide novel insights into the ultrasonographic findings and hoop function of the medial meniscus in patients with medial early KOA. Ultimately, our study demonstrates that it is possible to evaluate the hoop function of the medial meniscus using simple, dynamic ultrasonography and identify patients who require MRI.

Although we have made several efforts to minimize methodological limitations in this study, some limitations should be acknowledged. First, MRI was performed in mild knee flexion, and the examination position did not match the position used during ultrasonographic assessment. However, in this study, we observed a high correlation between ultrasonography and MRI measurements of MME, and we speculate that the effect on the results was small. In the future, measurements should be performed at the same knee position using both MRI and ultrasonography. Second, lower limb alignment was not measured in this study. Therefore, lower limb alignment may have differed significantly between the two groups. However, the difference in lower limb alignment was considered negligible because all participants had K–L grades of 0 or 1 with little deformation. Third, the study only included patients who had visited our clinic, which may have led to selection bias. Thus, it is possible that the period from the onset of symptoms and the degree of meniscal degeneration were not uniform among patients. In addition, we did not evaluate meniscal degeneration, which is considered to play a role in MME. Further studies involving multiple research centers are required to resolve these limitations. Fourth, a detailed analysis on medial meniscus posterior root tear such as the receiver operating characteristic (ROC) analysis, was not performed in this study to detect the cutoff value of medial meniscus posterior root tear because this study focused on medial meniscus posterior root tear and early KOA. However, this type of analysis is considered useful for ultrasonographic medial meniscus posterior root tear diagnosis in clinical situations. Therefore, we planned to focus on medial meniscus posterior root tear in future studies. A detailed analysis, such as an ROC analysis, will be performed in future studies. Finally, the evidence for setting MME > 2 mm as abnormal and MME > 1 mm as a decrease in the knee flexion angle is poor. Interestingly, one previous study reported that MME ˃3 mm is considered abnormal^31^. In contrast, even healthy adults have an MME of 0.9 ± 0.6 mm at a knee flexion angle of 0°^15^. In this study, abnormal MME was set at > 2 mm. We considered that the setting was reasonable because all participants had only early KOA, and there were few patients with MME > 3 mm under non-loading conditions. However, this is just our opinion, and we plan to include a control group with similar conditions in our future research. In addition, although there was no clear evidence for regarding MME ≥ 1 mm as a decrease in knee flexion, it was considered appropriate as a median value for dividing the two groups.

## Conclusions

In patients with early medial KOA, ultrasonography revealed a high rate (75%) of MME > 2 mm in the supine position. The ultrasonographic results for early medial KOA were in good agreement with MRI findings, and a lack of decrease in MME from 0° to 90° of knee flexion was a characteristic finding that suggested a loss of hoop function. These results indicate that it is possible to capture early medial KOA findings using ultrasonography, which can then be detected by MRI, implying that ultrasonography is a useful screening method for determining whether MRI should be performed in patients with early medial KOA.

## Methods

All procedures performed in studies involving human participants were in accordance with the ethical standards of the institutional and/or national research committee and with the 1964 Helsinki Declaration and its later amendments or comparable ethical standards. This retrospective study was approved by the Ethical Committee of Graduate School of Medical Sciences, Kanazawa University (approval no. 2726). Written informed consent was obtained from all patients included in this study.

**Figure 2:**
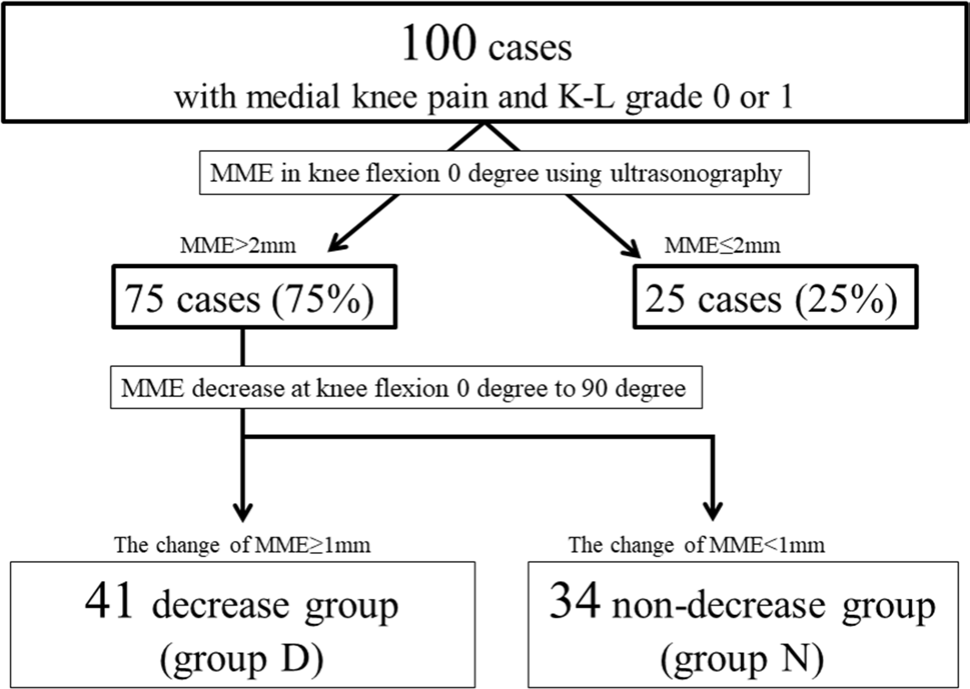
The flow chart of inclusion criteria and grouping in this study. *MME* medial meniscal extrusion.

Among all patients with medial knee pain examined between April 2018 and April 2019, the present study included 100 patients (100 knees) with a K–L grade of 0 or 1 in a standing anteroposterior X-ray view (Fig. 2). All patients were diagnosed with early medial KOA^32^. The selection was based on the following three criteria, similar to the study conducted by Luyten et al.^9^: Knee Injury and Osteoarthritis Outcome Score (KOOS) score ≤ 85% in at least two out of four categories, joint line tenderness or crepitus, and a K–L grade of 0 or 1. The other inclusion criteria were as follows: no locking or catching findings suggesting a symptomatic or traumatic meniscal tear in clinical examination; no history of ipsilateral knee surgery; no history of obvious traumatic accident, after which the knee pain started; no lateral pain in the knees or in other parts; ability to undergo MRI assessment; absence of inflammatory diseases, as observed by MRI evaluation; and no pacemakers, body piercings, or tattoos. Both ultrasonography and MRI were performed in all participants.

### MRI evaluation

**Figure 3:**
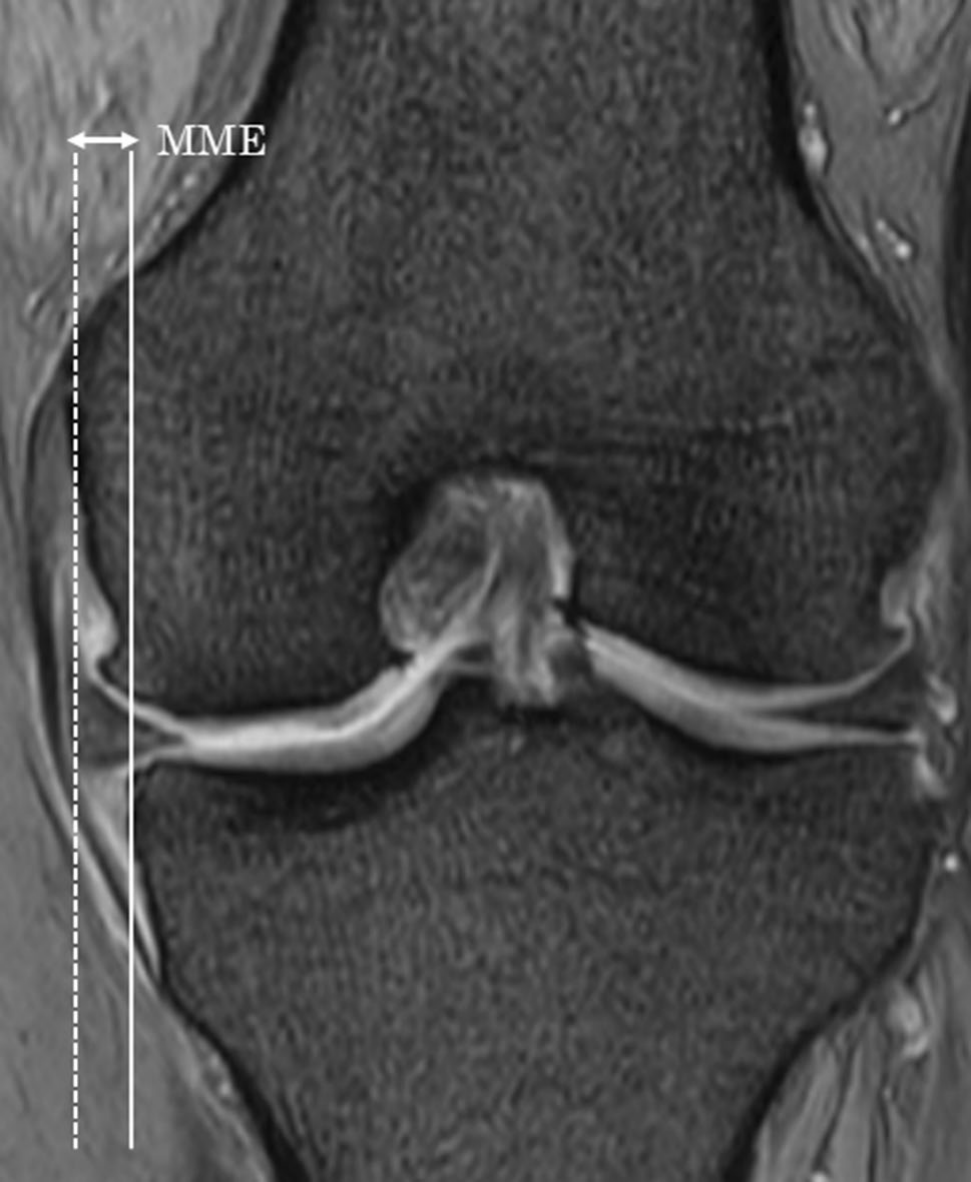
Methods for evaluating MME via coronal plane MRI. *MME* medial meniscal extrusion, *MRI* magnetic resonance imaging.

All participants underwent MRI at the first consultation. MRI was performed with mild knee flexion, and the MME and absence or types of medial meniscal tears were evaluated. A 1.5-T MRI machine (Singa HDxt; GE, Boston, MA) was used for image acquisition, with a slice thickness of 3 mm and a 1-mm interslice gap. These image data were analyzed using Synapse Vincent (Fuji Films, Tokyo, Japan). Fat -saturated proton density-weighted MR images were used to evaluate MME on the coronal plane, where the medial collateral ligament (MCL) is best depicted, and to evaluate the absence of types of medial meniscus tears on the coronal and sagittal planes. MME on MRI was defined as displacement from the margin of the tibial plateau and was measured as the distance (mm) between the margin of the tibial plateau and the peripheral border of the meniscal body (Fig. 3)^33^. The absence or types of medial meniscal tears were classified into five categories: no tear or only degenerative change, horizontal or flap tear, longitudinal tear, radial tear without posterior root, and posterior root tear. In addition, the absence of other inflammatory diseases was evaluated. To increase reliability, two experienced orthopedic surgeons who were blinded to patient information measured MME. The MRI findings were evaluated twice at intervals of about 2 weeks. The mean outcomes of the two assessors were accepted as the results. The intra-class correlation coefficient and inter-class reliability values were also calculated.

### Ultrasonographic evaluation

**Figure 4:**
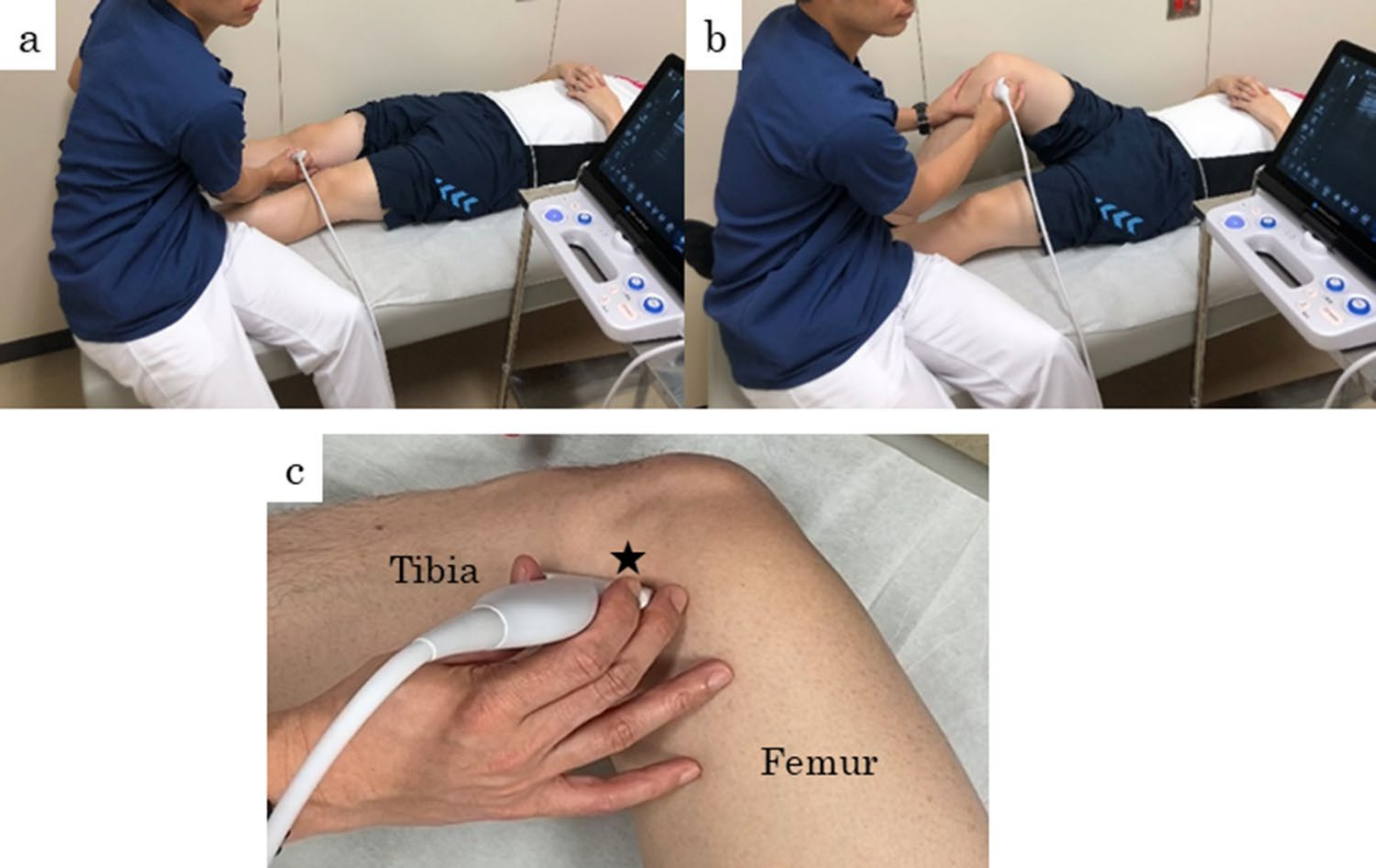
Ultrasonographic evaluations in the supine position at knee flexion angles of (**a**) 0° and (**b**) 90°. The appropriate probe position during MME evaluation (**c**). The femoral medial epicondyle was designated as a bony landmark (asterisk), and the proximal part of the probe was installed just behind it. *MME* medial meniscal extrusion.

**Figure 5:**
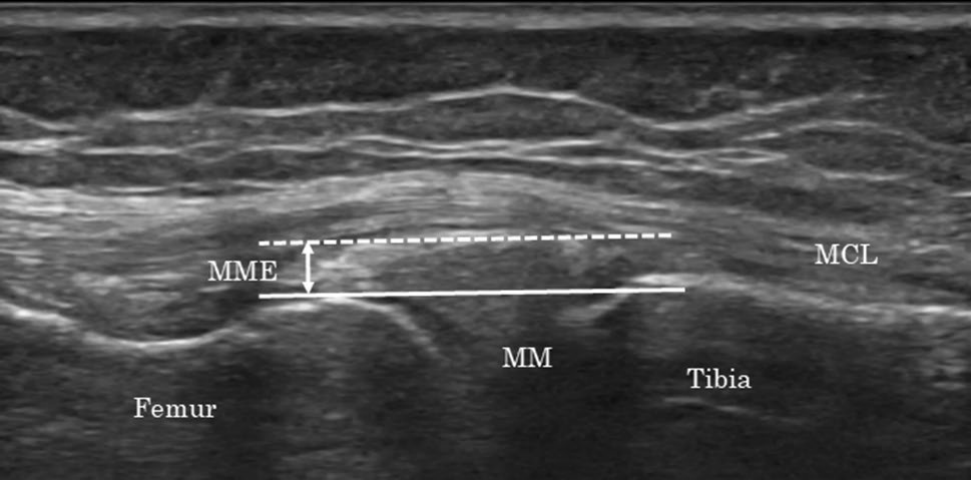
Methods for evaluating MME via ultrasonography using longitudinal sections parallel to the MCL. *MCL* medial collateral ligament, *MME* medial meniscal extrusion, *MM* medial meniscus.

**Figure 6:**
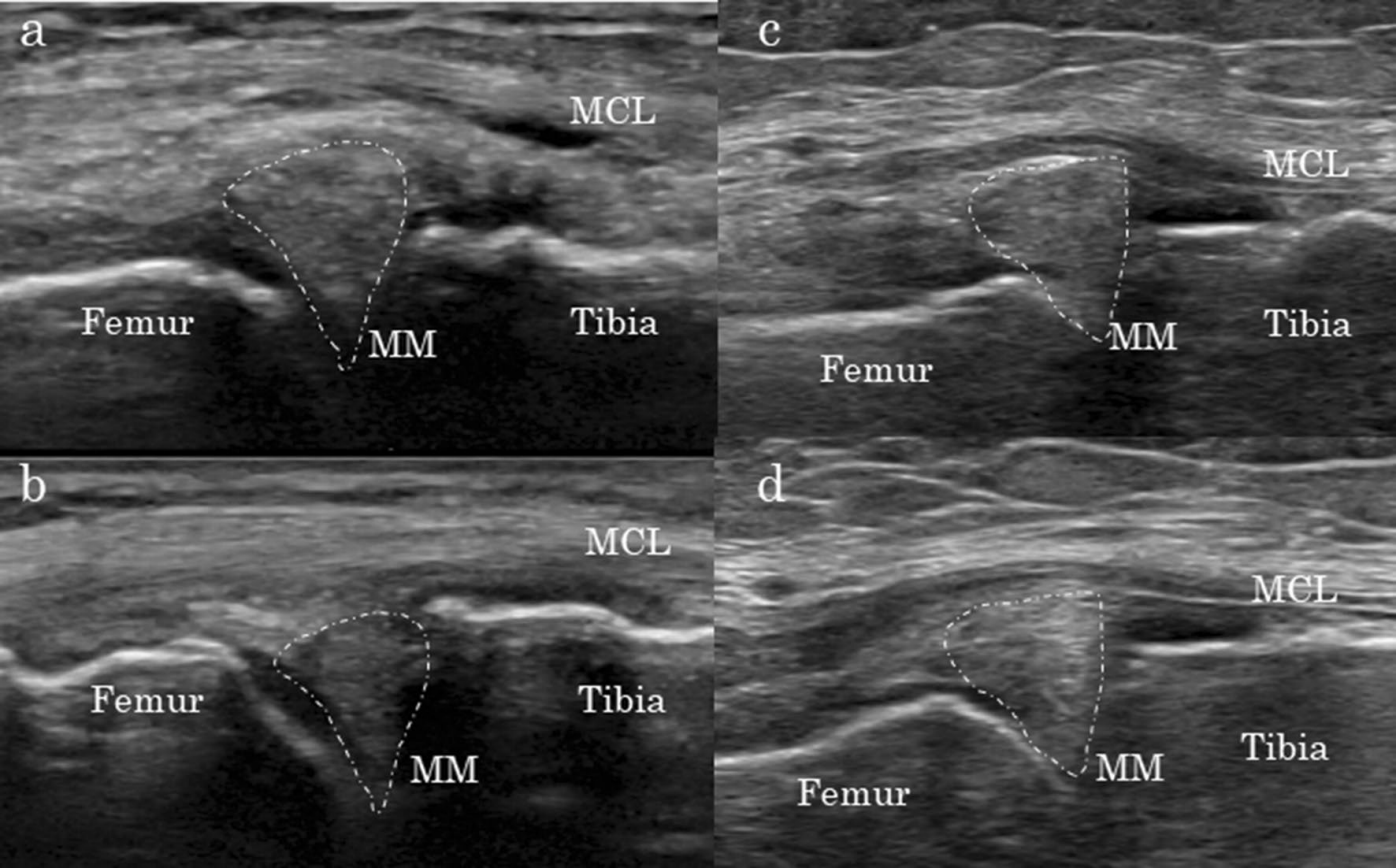
Difference in MME between 0° and 90° of knee flexion. Representative case in group D: (**a**) 0° and (**b**) 90°. Representative case in group N: (**c**) 0° and (**d**) 90°. The MME of each figure: (**a**) 3.5, (**b**) 0.5, (**c**) 3.6, and (**d**) 3.0 mm. MM is indicated by the dotted line. *MCL* medial collateral ligament, *MME* medial meniscal extrusion, *MM* medial meniscus.

During ultrasonography, MME at the affected knee with 0° and 90° of flexion was evaluated in the supine position (Fig. 4a,b) using the SNiBLE (KONICA MINOLTA, Tokyo, Japan) with an 18-MHz linear probe. Ultrasonographic images were obtained at the medial aspect of the knee, using longitudinal sections parallel to the MCL, where the MCL is best depicted (Fig. 5)^34^. Additionally, to ensure reproducibility of the ultrasonographic evaluation, the femoral medial epicondyle was designated as a bony landmark. Before the probe was installed, the femoral medial epicondyle was palpated, and the proximal part of the probe was installed just behind it (Fig. 4c). We were also mindful that the fat between the shallow and deep layers of the MCL was clearly visible and that the MCL was best depicted. Ultrasonographic images were saved as JPG files. Similar to the MRI evaluation, MME was defined as displacement from the margin of the tibial plateau and measured as the distance (mm) between the margin of the tibial plateau and the peripheral border of the meniscal body (Fig. 6)^17,35^ and was measured on the ultrasonography screen. An experienced orthopedic surgeon (KS) who was blinded to the MRI evaluation results performed all ultrasonographic medial meniscal assessments. Ultrasonographic MME findings were evaluated twice on the first visit day. The mean MME of the two assessments were accepted as the result, and intra-class correlation coefficients under knee flexions at 0° and 90° were also calculated.

In this study, MME > 2 mm as measured via ultrasonography at a knee flexion angle of 0° was defined as significant MME, as a previous ultrasonography study reported that even healthy adults have an MME of 0.9 ± 0.6 mm at a knee flexion angle of 0°^15^. Then, the patients were divided into two groups (Fig. 6): the decrease group (group D: Fig. 6a,b), in which MME decreased by > 1 mm at 90° of knee flexion, and the non-decrease group (group N: Fig. 6c,d).

### Statistical analysis

The data were analyzed using the Statistical Package for the Social Sciences for Windows (version 23.0; IBM Corp., Armonk, NY). The assessment items were analyzed using Student’s t-test between the two groups and the two-way analysis of variance for the change in MME from 0° to 90° knee flexion angles. The level of significance for all statistical analyses was set at α = 0.05. Age, sex, absence or type of meniscus tear, MME on ultrasonography, and MRI findings were compared between the two groups. The correlation between MRI measurements of MME and ultrasonography measurements of MME at 0° of knee flexion in the supine position was also examined using Pearson’s correlation coefficients (Evans’ boundaries) since these MME data were normally distributed (Shapiro–Wilk test). A prior power analysis for sample size was performed, which revealed that for an effect size of 0.6, power of 0.8, and α = 0.05, a total of 74 individuals were required.

## Data Availability

The datasets generated during and/or analyzed during the current study are available from the corresponding author on reasonable request.
